# Relationship between Gene Body DNA Methylation and Intragenic H3K9me3 and H3K36me3 Chromatin Marks

**DOI:** 10.1371/journal.pone.0018844

**Published:** 2011-04-19

**Authors:** Maria A. Hahn, Xiwei Wu, Arthur X. Li, Torsten Hahn, Gerd P. Pfeifer

**Affiliations:** 1 Department of Cancer Biology, Beckman Research Institute of the City of Hope, Duarte, California, United States of America; 2 Department of Molecular Medicine, Beckman Research Institute of the City of Hope, Duarte, California, United States of America; 3 Department of Information Sciences, Beckman Research Institute of the City of Hope, Duarte, California, United States of America; CNRS, France

## Abstract

To elucidate the relationship between intragenic DNA methylation and chromatin marks, we performed epigenetic profiling of chromosome 19 in human bronchial epithelial cells (HBEC) and in the colorectal cancer cell line HCT116 as well as its counterpart with double knockout of DNMT1 and DNMT3B (HCT116-DKO). Analysis of H3K36me3 profiles indicated that this intragenic mark of active genes is associated with two categories of genes: (i) genes with low CpG density and H3K9me3 in the gene body or (ii) genes with high CpG density and DNA methylation in the gene body. We observed that a combination of low CpG density in gene bodies together with H3K9me3 and H3K36me3 occupancy is a specific epigenetic feature of zinc finger (ZNF) genes, which comprise 90% of all genes carrying both histone marks on chromosome 19. For genes with high intragenic CpG density, transcription and H3K36me3 occupancy were not changed in conditions of partial or intensive loss of DNA methylation in gene bodies. siRNA knockdown of SETD2, the major histone methyltransferase responsible for production of H3K36me3, did not reduce DNA methylation in gene bodies. Our study suggests that the H3K36me3 and DNA methylation marks in gene bodies are established largely independently of each other and points to similar functional roles of intragenic DNA methylation and intragenic H3K9me3 for CpG-rich and CpG-poor genes, respectively.

## Introduction

Methylation of cytosines at promoters is an epigenetic mark, which is linked to gene silencing. The main regulatory function of DNA methylation has been associated with CpG-rich regions, CpG islands, which are frequently co-localized with gene promoters. It is well known that DNA methylation prevents binding of a number of transcription factors and attracts methylated-CpG binding domain proteins (MBDs) together with chromatin modifiers, which are responsible for the repressive chromatin structure [1]. In contrast to unmethylated promoters, repetitive elements are usually hypermethylated in normal cells whereas loss of repetitive DNA methylation has been linked to chromosomal instability and cancer [2], [3]. The mechanisms that control the inherently non-sequence-specific DNA (cytosine-5-)-methyltransferases (DNMTs) and establish a particular DNA methylation pattern along the genome are still unclear.

Connections between DNA methylation and chromatin modification processes have recently become apparent. Aberrant CpG island DNA methylation in cancer has been linked to the Polycomb-associated repressive histone mark, H3K27me3 [4], [5], [6]. This histone mark or its associated Polycomb components predispose target sequences to DNA methylation, for example during aging, inflammation and carcinogenesis [6], [7], [8], [9]. Di- and tri-methylated H3K9 (H3K9me2 and H3K9me3) are frequently found at aberrantly methylated gene promoters [5], [10]. It has been shown that H3K9 methylation promotes DNA methylation in *Arabidopsis thaliana*, in *Neurospora crassa* and in mammalian heterochromatin repeats [11], [12], [13]. Moreover, the H3K9 methyltransferase, SUV39H1 interacts with DNA (cytosine-5-)-methyltransferase 1 (DNMT1) and due to a common interaction partner, HP1, SUV39H1 can be found in the same complex with the *de novo* DNA methyltransferase, DNMT3B [12], [14], [15], [16]. Also, SETDB1, a methyltransferase that produces H3K9me3, has been detected in complex with the *de novo* DNA methyltransferase, DNMT3A [17].

Recently, a number of publications reported on the presence of extensive DNA methylation in gene bodies, downstream of transcription start sites (intragenic methylation). It was noticed that actively transcribed genes are associated with increased DNA methylation levels in *Arabidopsis thaliana* [18], [19] and in mammals [20], [21], [22]. In fact, in human cells most of the DNA methylation peaks are found in gene bodies rather than in promoters [22]. Inter- and intragenic CpG islands are preferentially susceptible to DNA methylation [23]. These findings indicate a possible new universal function of DNA methylation. Furthermore, it was observed that the transcription elongation mark, H3K36me3, is often correlated with DNA hypermethylation [24]. This histone modification is mainly established due to SETD2 histone methyltransferase activity [25], [26]. SETD2 forms a complex with hyperphosphorylated RNA polymerase II during elongation [26], [27]. The functional role of DNA methylation in transcription elongation processes or within gene bodies in general is unknown. It has been proposed that DNA methylation may inhibit initiation of aberrant transcription [28] and this function could be particularly important in gene bodies [18], [22]. A similar role is proposed for the H3K36me3 modification [29].

To more precisely understand the crosstalk between histone modifications, DNA methylation and transcription in mammalian cells, we profiled DNA methylation and chromatin modifications on human chromosome 19 in human bronchial epithelial cells (HBEC). To understand the impact of DNA methylation on chromatin and transcription, we performed a similar analysis in the HCT116 colon cancer cell line with double knockout of DNMT1 and DNMT3B, a cell line characterized by loss of 95% of DNA methylation [30]. To identify a role of H3K36me3 in establishing DNA methylation patterns in gene bodies, we analyzed epigenetic changes in cells lacking SETD2 after siRNA-mediated knockdown of SETD2.

## Results

### Crosstalk between histone modifications and DNA methylation on chromosome 19

To understand the interdependence between DNA methylation and histone modifications, we profiled DNA methylation patterns and several histone modifications along human chromosome 19 (chr19) on NimbleGen tiling arrays in human bronchial epithelial cells immortalized with hTERT and CDK4 [31]. Chromosome 19 was chosen because of its high gene density and CpG methylation frequency [22]. These data sets were compared to expression profiles obtained on the Affymetrix platform. DNA methylation patterns were analyzed by using two complementary methods, the UnMethylCollector™ (UMC) method, here applied for the first time to genome-wide profiling of unmethylated CpGs on tiling arrays, and the methylated-CpG island recovery assay (MIRA). Whereas UMC enriches the unmethylated DNA fraction of the genome due to the high affinity of the CXXC domain of the MBD1 protein to unmethylated CpGs [23], [32], MIRA is used to enrich methylated genomic DNA fragments by using the protein complex of MBD2B and MBD3L1 [22], [33]. The combination of both assays allows us to comprehensively map DNA methylation patterns along chromosomes. These methylation patterns were then compared to H3K27me3, H3K9me3, H3K9/K14 acetylation and H3K36me3 histone modification profiles.

**Figure 1:**
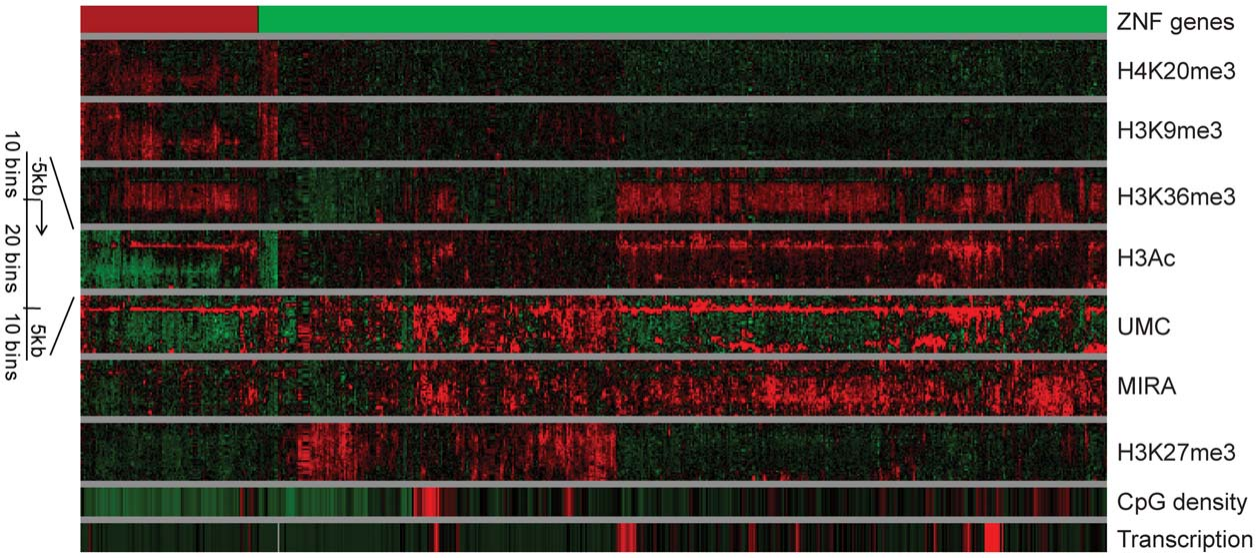
Clustering analysis of epigenetic profiles of genes located on chr19. Individual genes are indicated by single vertical lines depicting the epigenetic status from the 5' end (top) to the 3' end (bottom) of each gene. Each gene body was divided into 20 bins. The 5 kb upstream of the TSS and 5 kb downstream of the 3' gene end were divided into 10 bins. The average signal for each single bin is indicated. Green, red and black colors represent low, high and average occupation by epigenetic marks in comparison to the average signal on the chromosome, respectively. Zinc finger (ZNF) genes represent a unique epigenetic class.

**Figure 2:**
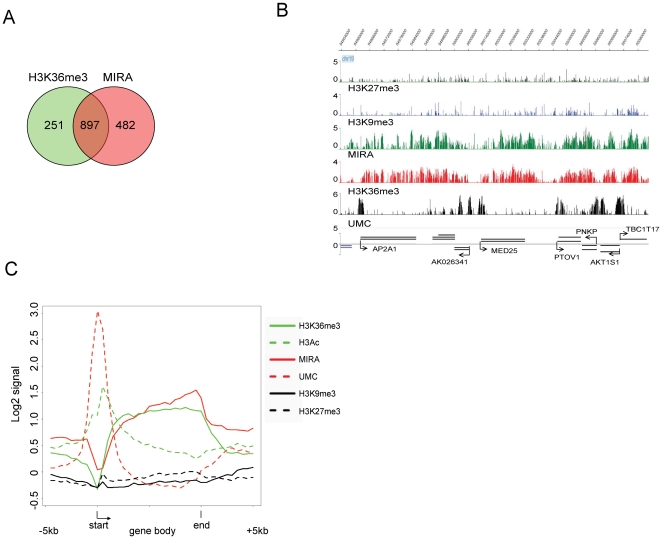
Crosstalk between DNA methylation and chromatin modifications on chr19 in human bronchial epithelial cells. **A**. Gene bodies are marked by DNA methylation and H3K36me3 on human chr19 in HBEC. The numbers of genes with gene bodies marked by H3K36me3, DNA methylation or both, with at least 20% of gene body length covered by the respective mark, are indicated (p<0.001; Chi square test). **B**. Representative epigenetic profile of genes harboring DNA methylation and H3K36me3 in the gene body. Signals plotted as negative log10(p-values) are shown for the chromatin marks H3K9me3, H3K27me3 and H3K36me3. Methylated CpG density mapped by MIRA is shown along with the mapping of unmethylated CpGs by UnmethylCollector (UMC) **C**. Composite profile of genes with gene bodies marked by H3K36me3 and DNA methylation over at least 20% of gene body length. Each gene body was divided into 20 bins. Sequences up to 5 kb upstream of the TSS and 5 kb downstream of the 3' gene end were divided into 10 bins. Average of occupation for each single bin is indicated. The chromatin marks histone H3 acetylation (K9/K14), H3K9me3, H3K27me3 and H3K36me3 were analyzed along with methylated CpGs (MIRA) and unmethylated CpGs (UMC).

The distribution of epigenetic marks over all genes of chr19 (Figure 1) showed that the DNA methylation signal (MIRA) generally is opposite to the unmethylated CpG (UMC) signal (see also Figure S1G). For most genes, DNA methylation also inversely correlates with the repressive histone marks H3K9me3 and H4K20me3. It is apparent that the DNA methylation level is often high in genes showing H3K36me3 enrichment consistent with previous results [20], [22], [24]. Analysis of signals within gene bodies of chr19 genes indicates co-localization of DNA methylation and H3K36me3 (Figure 1; Figure 2A,B,C). Gene body was defined as the entire gene from the transcription start site to the end of the transcript. Absence of H3K36me3 was associated with partial methylation or hypomethylation status of gene body DNA as indicated by UMC signal (Figure 1). Genes carrying both intragenic DNA methylation and H3K36me3 are sharply unmethylated at the promoter and hypermethylated within the gene body (Figure 1; Figure 2B,C). They also show acetylated histone H3 near their 5′ ends (Figure 1). At the 3′ end of genes, in H3K36me3-free areas, we observed a loss of DNA methylation by using both methods, MIRA and UMC (Figure 1; Figure 2B,C). This finding was confirmed by combined bisulfite restriction analysis (COBRA) for the *LSM4* gene (Figure S1A). These observations indicate a strong link between H3K36me3 and DNA methylation since absence of H3K36me3 is immediately reflected in reduction of DNA methylation. We further validated the correlation between H3K36me3 and gene body DNA methylation with additional COBRA methylation assays for the *POLD1* and *NFKBIB* genes (Figure S1B,C).

The plotting of unmethylated DNA peaks mapped by UMC versus histone H3 acetylation and H3K36me3 modifications showed that unmethylated DNA is enriched for H3 acetylation but is reverse to H3K36me3 (Figure 2C). The profiling of other epigenetic marks indicated that genes carrying H3K36me3 and DNA methylation in the gene body contain very low levels of H3K27me3, H3K9me3 and H4K20me3 (Figure 1; Figure 2B,C). We then performed correlative analysis of DNA methylation and H3K36me3 signals within gene bodies (Figure S1D,E). For these genes, plotting the MIRA signal in gene bodies versus H3K36me3 showed a Pearson correlation coefficient of R = 0.38 (p<0.001) whereas the UMC signal was negatively correlated with H3K36me3 (R = -0.39; p<0.001). The data sets suggest that presence of H3K36me3 is associated with an increase of DNA methylation in gene bodies and absence of H3K36me3 is associated with reduced levels of methylated DNA. Histone H3 acetylation or unmethylated CpGs were also negatively correlated with the MIRA signal (Figure S1F,G; P<0.00001).

Clustering analysis of epigenetic profiles of gene bodies showed that a subset of gene-specific DNA methylation patterns generated by MIRA is also associated with the Polycomb mark H3K27me3 (Figure 1; Figure S2A). H3K27me3-covered genes are repressed according to mRNA expression data and characterized by absence of the H3K36me3 mark and by the presence of both MIRA and UMC signals along the gene body (Figure 1; Figure S2B,C). Comparison of the two gene groups, genes covered by H3K27me3 and by DNA methylation covering at least 20% of gene body length and genes harboring H3K36me3 and DNA methylation over at least 20% of gene body length, showed that the DNA methylation level is higher in genes with H3K36me3 and DNA methylation than in genes with H3K27me3 and DNA methylation and both groups are characterized by equal CpG density in gene bodies (Figure S2D,E).

### Gene bodies of zinc finger genes are enriched in H3K9me3 and H3K36me3

**Figure 3:**
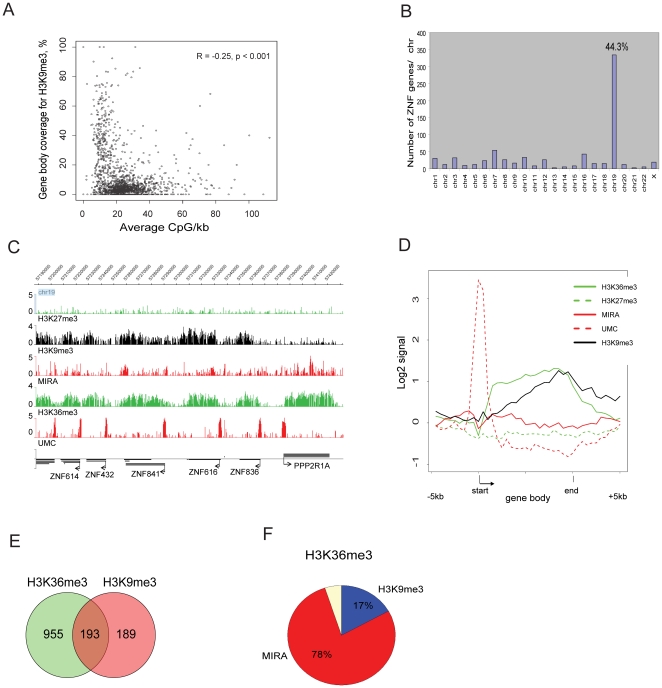
Epigenetic profile of ZNF genes on chr19 in human bronchial epithelial cells. **A**. Genes located on chr19 sorted by CpG density in gene bodies and by percentage of gene body length covered by H3K9me3. **B**. Distribution of ZNF genes in the human genome. The number of ZNF genes on individual chromosomes is shown. **C**. Representative epigenetic profile of ZNF genes. Gene names, directions of transcription and gene coordinates on the chromosome are indicated. Note the simultaneous occupation of ZNF gene bodies by H3K9me3 and H3K36me3. **D**. Composite profile of genes with gene bodies marked by H3K36me3 and H3K9me3 over at least 20% of gene body length. Each gene body was divided into 20 and the 5 kb upstream of the TSS and 5 kb downstream of the 3' gene end were divided into 10 bins. The average signal for each single bin is indicated. **E**. Gene bodies marked by H3K9me3 and H3K36me3 on human chr19 in HBEC. The number of genes with gene bodies marked by H3K36me3 or H3K9me3 or by both marks over at least 20% of gene body length is shown (p = 0.02; Chi square test). Ninety percent of the dual-occupied genes are zinc finger genes. **F**. Genes marked by H3K36me3 on chr19 in HBEC. The diagram represents the distribution of H3K9me3 and DNA methylation in gene bodies containing H3K36me3. Genes were assumed to carry a specific modification if at least 20% of gene body length was covered by the analyzed mark. Co-occupancy of H3K9me3 and H3K36me3 is a hallmark of ZNF genes.

The H3K9me3 profile showed that this epigenetic mark strongly co-localizes with the other repressive mark, H4K20me3 (Figure 1). Similar to the group of H3K27me3-covered genes, genes associated with H3K9me3 have low levels of transcription. H3K9me3-covered gene bodies are generally CpG poor (Figure 3A). Only occasional spots of H3K9me3 enrichment can be found in gene bodies with average or high CpG density. Gene bodies of H3K9me3-associated genes have low levels of transcription, and either low CpG content or low levels of DNA methylation as indicated by a UMC signal (Figure 1). Analysis of DNA methylation status by COBRA confirmed the presence of unmethylated DNA or partially methylated DNA in gene bodies of several H3K9me3-covered genes, including *NALP11*, *UNC13A* and *ZNF563* (Figure S3A). Analysis of the H3K9me3-covered genes showed that these genes are characterized by specific zinc finger motifs according to DAVID analysis (Figure S3B) [34]. Strikingly, from 382 genes covered by H3K9me3 on chr19, 62% are zinc finger (ZNF) genes (Figure 1). Chr19 carries the highest number of ZNF genes in comparison to the other human chromosomes (Figure 3B). Forty-four percent (334 of 755) of all ZNF genes are located on chr19.

It is well known that H3K9me3 is a heterochromatin repressive mark. However, we found that some genes in H3K9me3 blocks harbor also the H3K36me3 modification typical of transcribed genes (Figure 1; Figure 3C,D). Ninety percent of the 193 dual-occupied genes are zinc finger protein encoding genes (Figure 3E). Most of the ZNF genes carry H3K36me3 in bronchial epithelial cells but they are characterized by a low level of expression in comparison to other genes (Figure 1). This level of transcription is comparable to that of H3K27me3-repressed genes. However, in contrast to H3K27me3-associated genes, ZNF genes are occupied by H3K36me3. ZNF gene promoters are frequently associated with high CpG density sequences (CpG islands), in contrast to their gene bodies, which have low CpG density. For most active ZNF genes, promoters are marked by unmethylated DNA according to the UMC and MIRA profiles and by histone acetylation (Figure 1; Figure 3C,D). We did not observe any H3K27me3 enrichment in gene bodies of ZNF genes.

Our data indicate that 78% of all genes with the H3K36me3 mark are associated with intragenic DNA hypermethylation whereas 17% of the H3K36me3-marked genes carry H3K9me3 in their gene body (Figure 3F). Based on our criteria for gene coverage, these numbers add up to almost 100% for all H3K36me3-associated genes on chr19 and are indicating possible links between high CpG density and DNA hypermethylation in one class of genes and between low CpG density and H3K9me3 in the gene bodies of the other class of genes.

The relationship between H3K36me3 and DNA methylation and between H3K36me3 and H3K9me3 was further analyzed within genes of different expression levels and with respect to exon-intron structure (Figure S4). Expression data from Affymetrix exon arrays were used in this analysis. Again, we subdivided the genes into two categories: genes covered by H3K36me3 and CpG methylation (MIRA) (Figure S4A,B) and genes covered by H3K36me3 and H3K9me3 (Figure S4C,D). These data show that the exons of the more highly expressed genes carry higher levels of H3K36me3 for both categories of genes. On the other hand, the MIRA signal is stronger for genes with higher expression levels while the H3K9me3 signal does not correlate with expression levels in the other category of genes. When examining all exons combined, we did not find much difference between exons and introns for genes co-occupied either by DNA methylation (MIRA signal) and H3K36me3 (Figure S4A,B) or by H3K36me3 and H3K9me3 (Figure S4C,D) although the H3K9me3 signal was slightly lower in introns. We also looked at DNA methylation, H3K36me3 and H3K9me3 at the first and last exons and at the first and last coding exons, respectively, in genes with low and high expression levels, respectively (Figure S4). All three modifications were generally lower at the first exons but increased at the first coding exons. We think this is because many genes have 5′ untranslated first exons, which often fall within the CpG islands near their promoters. CpG islands are generally devoid of these repressive modifications.

### DNA methylation and H3K36me3 histone marks in HCT116 cells deficient in DNMT1 and DNMT3B

Since our data indicated a strong link between DNA methylation and the H3K36me3 mark, we investigated epigenetic profiles in the HCT116 colorectal cancer cell line and in its isogenic counterpart with double knockout of DNMT1 and DNMT3B [30]. As reported, 95% of DNA methylation is lost in this cell line. By using the COBRA method, we confirmed almost complete loss of DNA methylation in repetitive LINE1 elements in HCT116-DKO cells in comparison to the original cell line (Figure S5A). We profiled DNA methylation, H3K9me3, H3K36me3 and transcripts (by cDNA hybridization) in HCT116 and HCT116-DKO cells along chr19. These data were also compared to results from Affymetrix exon arrays. To evaluate the epigenetic changes in the *DNMT1* gene itself caused by disruption of exons 3-5, we examined the epigenetic profile of the *DNMT1* locus located on chr19 (Figure S5B). We identified H3K36me3 occupancy in HCT116-DKO cells with loss of signal for the deleted exons 3 to 5. Also, analysis of Affymetrix exon arrays indicated a 2.5-times reduced level of DNMT1 transcription. These data confirmed the gene deletion at the *DNMT1* gene in these cells and suggest reduced levels of a transcript presumably encoding a truncated DNMT1 protein [35], [36].

**Figure 4:**
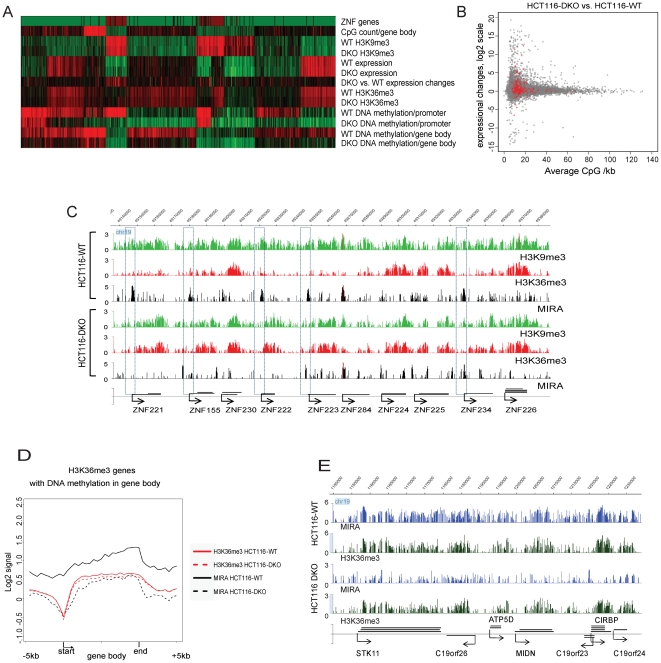
Epigenetic changes in HCT116-DKO cells. **A**. Clustering analysis of epigenetic changes on chr19 in HCT116-DKO cells in comparison to HCT116-WT cells. Each vertical line represents one gene. Green, red and black colors represent low, high and average occupation by epigenetic marks, respectively, in comparison to the average signal along the chromosome. Similarly, gene expression is indicated by green, red and black color showing low, high and average expression level, respectively, in comparison to average transcription levels on chr19. For expression changes, red, green and black colors represent activation, repression and no change of transcription activity, respectively, in HCT116-DKO cells in comparison to HCT116-WT cells. Abbreviations WT and DKO are for HCT116-WT and HCT116-DKO cells, respectively. **B**. Interrelation between genome-wide expression changes in HCT116-DKO cells in comparison to HCT116-WT cells and gene body CpG density (R = -0.02, p  =  0.03). Red color indicates ZNF genes. **C**. Representative epigenetic profiles of activated ZNF genes in HCT116-DKO and silent state of these genes in HCT116-WT cells. Direction of transcription and gene coordinates on chromosome 19 are indicated. The boxes span promoters of genes that become demethylated and reactivated in DKO cells as indicated by appearance of the H3K36me3 mark in their gene bodies. **D**. Composite profile of H3K36me3-associated genes in HCT116-DKO and in HCT116-WT cells. The profile was created for genes with gene bodies covered by H3K36me3 and DNA methylation with at least 20% of gene body length covered in HCT116-WT cells. Each gene body was divided into 20 bins and the 5 kb upstream of the TSS and 5 kb downstream of the 3' gene end were divided into 10 bins. The average signal for each single bin is indicated. **E**. H3K36me3 is not lost in regions with extensive loss of DNA methylation in gene bodies. Despite of loss of DNA methylation (blue tracks) in gene bodies, the H3K36 profile (black tracks) is almost unaltered.

DNA methylation profiles of chr19 for HCT116-WT and HCT116-DKO cells confirmed reduction of DNA methylation in DKO cells in promoters and in gene bodies (Figure 4). However, we observed that some genes harboring H3K36me3 retained substantial DNA methylation levels in the gene body in the DKO cell line (Figure 4A,D), perhaps related to activity provided by DNMT3A, which recently has been linked to intragenic DNA methylation [37]. Specific knockout lines for DNMT3A are not available and our attempts to knock down DNMT3A using siRNA were not successful. For a small group of active genes, DNA methylation was almost not affected in the gene body in HCT116-DKO cells. These data were confirmed for the *POLD1* and *NFKBIB* genes by COBRA, which showed remaining DNA methylation in gene bodies (Figure S5C). DNA methylation analysis in promoter areas indicated that a group of promoters drastically lost DNA methylation in HCT116-DKO cells (Figure 4A,C). However, this fact only infrequently correlated with transcriptional activation. Importantly, for genes with substantial gene body DNA methylation, transcription and H3K36me3 occupancy were not changed in condition of partial or intensive loss of DNA methylation along the gene bodies in the HCT116-DKO cell line (Figure 4A,D,E).

We performed composite profile analysis of H3K36me3 and DNA methylation for the group of genes, which carry both of these marks in HCT116-WT cells (Figure 4D). This profile indicated that DNA methylation mimics H3K36me3 over gene bodies in HCT116-WT and in HCT116-DKO cells although DNA methylation levels are reduced in DKO cells. Also, we observed intense DNA methylation around many more transcription start sites in HCT116-WT cells in contrast to immortalized HBEC (Figure 2C and Figure 4D). Double knockout of DNMT1 and DNMT3B resulted in a loss of DNA methylation around promoters and in a decrease of DNA methylation levels in gene bodies (Figure 4C,D). It is important to point out that H3K36me3 levels were not specifically affected in genes with intensive loss of DNA methylation in the gene body (Figure 4D,E).

### ZNF genes become activated due to reduction of DNMT activities

According to data on transcriptional changes obtained from Affymetrix exon arrays, almost only ZNF genes became activated on chr19 in the HCT116-DKO cell line (Figure 4A). This fact was associated with *de novo* H3K36me3 occupation of gene bodies and promoter DNA demethylation in HCT116-DKO cells (Figure 4C). Since ZNF genes are localized in H3K9me3-occupied areas in HBEC, we profiled this mark in HCT116-WT and HCT116-DKO cells. We observed that activation of ZNF genes is associated with a reduction of the H3K9me3 mark in promoter areas and in gene bodies (Figure 4C). Further analysis indicated that *de novo* H3K36me3 occupation in HCT116-DKO cells is typical for low CpG density gene bodies. Since H3K36me3 is highly correlated with transcriptional activity, we plotted transcription activity changes in HCT116 DKO relative to WT cells versus CpG density in gene bodies, genome-wide, by using results from Affymetrix exon arrays (Figure 4B). Our data indicate that gene activation occurs more frequently than silencing in HCT116-DKO cells. Transcriptional upregulation in HCT116-DKO versus HCT116-WT cells was almost specific for genes with low CpG density in gene bodies. Also, the level of transcriptional change is gradually decreasing with increase of CpG density in gene bodies (Figure 4B). These data are correlated with relatively rare changes in the H3K36me3 profile in HCT116-DKO cells and infrequent transcriptional changes observed on chr19 (Figure 4A). We performed composite profile analysis for activated genes bearing H3K9me3 in gene bodies in HCT116-DKO cells (Figure S6A). These data confirmed our observation about the mechanism of ZNF gene activation and showed that gene activation of H3K9me3-covered genes is associated with an increase of H3K36me3 in the gene body, a decrease of H3K9me3 in the promoter and gene body, and loss of DNA methylation at the promoter. On the other hand, repression of H3K9me3-covered genes is associated with a slight increase of H3K9me3 level at the promoter area and loss of H3K36me3 (Figure S6B).

### DNA methylation is not affected by reduction of H3K36me3 levels

**Figure 5:**
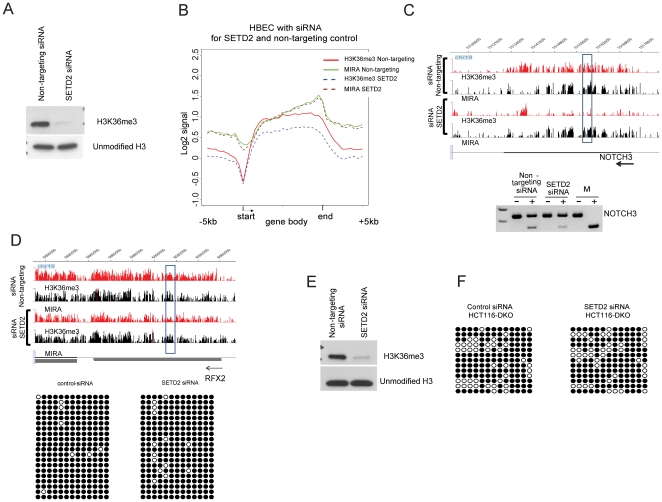
Epigenetic changes after SETD2 knockdown. **A**. Western blot for HBEC cells after non-targeting siRNA and SETD2 siRNA transfection using anti-H3K36me3 antibodies and unmodified histone H3 antibodies. **B**. Composite profile of epigenetic changes after SETD2 siRNA knockdown in HBEC. The profile was created for gene bodies marked by H3K36me3 and DNA methylation over at least 20% of gene body length. Each gene body was divided into 20 bins and the 5 kb upstream of the TSS and 5 kb downstream of the 3' gene end were divided into 10 bins. The average signal for each single bin is indicated. **C**. Analysis of DNA methylation in the gene body of the *NOTCH3* gene in conditions of H3K36me3 deficiency. The H3K36me3 profile of *NOTCH3* after non-targeting siRNA and SETD2 siRNA transfections in HBEC cells is shown. The region analyzed by COBRA methylation assays is indicated by a box. Using gene-specific primers, bisulfite-converted DNA was amplified. After cutting with HpyCH4IV recognizing CpG dinucleotides, mock (-) and enzyme-digested (+) PCR products were fractionated on a 2% agarose gel. In vitro CpG-methylated human DNA (M) served as a positive control. Cleavage indicates DNA methylation. **D**. Analysis of H3K36me3 and DNA methylation in the gene body of *RFX2* in conditions of H3K36me3 deficiency. The region analyzed by bisulfite sequencing is marked by a box. Using gene-specific primers, bisulfite-converted DNA was amplified, cloned and 20 individual clones were sequenced. White circles, unmethylated CpG sequences; black circles, methylated CpG sequences. **E**. Western blot for HCT116-DKO cells after non-targeting siRNA and SETD2 siRNA transfection using anti-H3K36me3 antibodies and unmodified histone H3 antibodies. **F**. DNA methylation analysis of the gene body of *NOTCH3* after non-targeting siRNA and SETD2 siRNA transfection of HCT116-DKO cells. Using gene-specific primers, bisulfite-converted DNA was amplified, cloned and 13 individual clones were sequenced. White circles, unmethylated CpG sequences; black circles, methylated CpG sequences.

To understand if the H3K36me3 mark plays a role in the establishment and maintenance of intragenic DNA methylation patterns, we decreased the level of this chromatin modification in cells to monitor possible changes of DNA methylation and transcriptional activity. Towards this goal, we performed transfection experiments with siRNA for SETD2, the major H3K36me3 methyltransferase [25], in HBEC cells and in HCT116-DKO cells. According to our Western blotting data, H3K36me3 levels were drastically reduced in SETD2 siRNA treated HBEC in comparison to non-targeting siRNA transfected cells (Figure 5A). Expression analysis by Affymetrix exon arrays indicated that 304 genes underwent transcriptional changes due to SETD2 siRNA treatment. Of these, 209 genes were significantly repressed due to SETD2 siRNA transfection. From these genes, 17 genes were localized on chr19 including 10 ZNF genes. Genome-wide, transcription of 20 ZNF genes was affected due to SETD2 knockdown usually showing repressed transcriptional activity. Since a much higher number of genes sustain a decrease of H3K36me3 levels according to ChIP on ChIP data than the number of genes showing transcriptional changes in SETD2 siRNA experiments, we assume that the H3K36me3 mark is not chiefly involved in maintaining transcript levels.

We performed composite profile analysis of gene bodies in SETD2 siRNA treated cells versus controls (Figure 5B). These data confirmed a reduction of H3K36me3 levels after siSETD2 treatment. Our data did not show a reduction of DNA methylation levels in gene bodies. Importantly, we verified the DNA methylation status for several genes with substantial loss of H3K36me3 levels in gene bodies according to the microarray results. Our COBRA analysis and bisulfite sequencing did not identify a reduction of DNA methylation after siRNA for SETD2 versus non-targeting siRNA (Figure 5C,D) (p = 0.276; Fisher's exact test, two-tailed, for bisulfite data). Further, we tried to identify a possible role of H3K36me3 under conditions of DNMT deficiency in HCT116-DKO cells. Similar to HBEC, we treated HCT116-DKO cells with SETD2 siRNA for five days. This experiment resulted in significant loss of H3K36me3 (Figure 5E). However, we could not observe a reduction of DNA methylation in SETD2 siRNA treated cells (Figure 5F) (p = 1.000; Fisher's exact test, two-tailed). Based on these observations, we conclude that DNA methylation patterns in gene bodies are established independently of the H3K36me3 mark.

## Discussion

In our study, we observed that the H3K36me3 mark reflects active transcription. These results support previous genome-wide mapping data of this histone modification, observations of H3K36me3 as a mark for the transcription elongation process and direct interaction of SETD2 with hyperphosphorylated RNA polymerase II [25], [38], [39]. According to our detailed analysis of chromosome 19, H3K36me3 presence is clearly correlated with DNA methylation levels in gene bodies. Similar results reporting correlations of H3K36me3, transcript levels, and gene body DNA methylation were obtained in IMR90 cells, in human embryonic stem cells and in human B-lymphocytes [20], [22], [24]. Our results also indicate that absence of H3K36me3 is associated with partial methylation or hypomethylation status of gene body DNA (Figure 1).

The correlation between DNA methylation and H3K36me3 in gene bodies was also investigated in HCT116-DKO cells lacking DNA methyltransferases. In HCT116-DKO cells, we observed a slight reduction of total H3K36me3 levels compared to HCT116-WT cells but independent of DNA methylation changes. This observation can be explained by the fact that H3K36me3 levels are associated with the cell cycle and that HCT116-DKO cells proliferate more slowly than HCT116-WT cells [40], [41]. However, we did not observe any substantial loss of H3K36me3 in genes with intensive loss of DNA methylation in HCT116-DKO cells (Figure 4). Reduction of H3K36me3 levels by siRNA-mediated knockdown of SETD2 rarely affected transcriptional activity (only 304 genes genome-wide were significantly affected in these experiments). H3K36me3 loss failed to reduce DNA methylation in gene bodies in both HBEC and HCT116-DKO cells (Figure 5). These data suggest that DNA methylation and H3K36me3 are not associated directly and that both marks are established independently during the transcription elongation process. Our data are not directly consistent with recent observations showing an interaction of the PWWP domain of DNMT3A with the H3K36me3 modification [42]. In our hands, the loss of H3K36me3 did not result in noticeable reduction of DNA methylation in HBEC or in HCT116-DKO cells. Since presence of active DNMT1 and DNMT3B in HBEC could make it difficult to detect a loss of DNA methylation caused by absence of H3K36me3, we performed SETD2 siRNA experiments also in HCT116-DKO cells lacking both DNMT1 and DNMT3B but retaining DNMT3A. We assumed that an effect of H3K36me3 on DNMT3A and DNA methylation would be enhanced in conditions of absence DNMT3B and in presence of reduced levels of truncated DNMT1. However, reduction of H3K36me3 in these cells did not result in loss of DNA methylation either. Nonetheless, it is possible that the low amounts of H3K36me3 remaining after SETD2 knockdown may still suffice for maintaining DNA methylation.

Since gene body DNA methylation and H3K36me3 are correlated, yet not directly dependent on each other, we assume that both marks are established independently during the transcription elongation process. SETD2 is a component of transcription elongation complexes and binds to RNA polymerase II [26], [27]. Whether the DNA methylation machinery is similarly connected to elongating RNA polymerase II complexes remains to be determined but there is evidence from plants describing proteins that interact with RNA polymerase II and function in RNA-directed DNA methylation [43], [44].

We found that H3K9me3 is mostly associated with genes characterized by low CpG density in the gene body. The reason for the association of low CpG density genes with H3K9me3 is unclear. We assume that genes with low CpG density in the gene body have a specific epigenetic regulation. In HCT116-DKO cells, almost only low CpG density genes became activated in conditions of DNMT deficiency. It appears that genes having high levels of CpG methylation in both the promoter and the gene body are difficult to reactivate by loss of DNA methylation. Similar to DNA methylation, which is present in gene bodies of many genes, the H3K9me3 mark covers preferentially ZNF genes intragenically. The functional role of the H3K9me3 histone modification in gene bodies is currently unknown, as is the function of DNA methylation in gene bodies. Like DNA methylation, H3K9me3 is generally considered a repressive mark and is usually associated with compacted chromatin. It was suggested that DNA methylation in gene bodies may block false initiation of transcription [18], [22]. We propose that H3K9me3 may play a similar role in gene bodies and replaces the intragenic function of DNA methylation under conditions of low CpG density. Unlike genes with intragenic DNA methylation, which are associated with intermediate to high levels of transcription [22], genes with H3K9me3 in the gene body are associated with very low levels of transcription. We suppose that H3K9me3 may slow down RNA polymerase II much more effectively than DNA methylation in gene bodies. This fact may be reflected in low levels of ZNF transcripts.

We observed that ZNF genes are characterized by H3K9me3 and H3K36me3 dual occupation and by very low CpG density in gene bodies. This specific feature was associated with activation of this group of genes in conditions of DNMT deficiency. ZNF genes were previously separated as a group of genes associated with H3K9me3 in the promoter [24]. Also, the regulation of ZNF genes was linked to the KAP1 factor, which is mostly associated with 3' gene ends and repression of ZNF genes [46], [47]. These facts also indicate ZNF genes as a separate epigenetic group. The observations suggest that functionally related genes can be divided into groups with specific epigenetic marks.

In conclusion, the two main findings of our study are that (i) the H3K36me3 and DNA CpG methylation patterns in gene bodies are not directly dependent on each other, and (ii) from their CpG density-dependent gene-specific localization patterns, we suggest that there is a similar functional role for intragenic DNA methylation and intragenic H3K9me3 for CpG-rich and CpG-poor genes, respectively. Future studies will determine whether the role of these two intragenic repressive marks indeed lies in suppression of spurious intragenic gene transcription as is assumed by some current models or whether they have other functional roles that are currently not anticipated.

## Materials and Methods

### Tissue culture, siRNA transfection and Western blot

HCT116-WT and HCT116-DKO (DKO1 line) cells were generous gifts from B. Vogelstein (Johns Hopkins University) [30]. These cells were cultured in McCoy's 5A medium with 10% FBS. Bronchial epithelial cells immortalized with hTERT and Cdk4 were kindly provided by John D. Minna (The University of Texas Southwestern Medical Center). The immortalization and culturing conditions for HBEC have been described previously [31].

For siRNA treatment, cells were transfected twice, 96 hours apart, each transfection using Lipofectamine 2000 (Invitrogen) and OptiMEM® I medium (Invitrogen) according to the manufacturers' instructions. For the *SETD2* gene, we used siRNA D-012448-03, HYPB (Dharmacon). For non-targeting control, siRNA D-001810-01-05 was used (Dharmacon).

For protein analysis, the same number of cells for control and *SETD2* siRNA experiments were used. Samples were denatured in Laemmli sample buffer (BioRad; Hercules, CA) and resolved in pre-cast gels (BioRad). After semi-wet transfer, membranes were blocked with 5% milk (BioRad) in PBS with 0.1% Triton X-100 and further incubated with specific antibodies. We used the following antibodies for Western blotting: H3K36me3 (Abcam and SA Bioscience), histone H3 (Abcam), and beta-actin (Sigma). Detection was achieved using HRP-conjugated secondary antibodies (BioRad) and ECL Western Blotting System (GE Healthcare).

### Expression analysis

Total RNA was isolated with the RNAeasy kit (Qiagen; Valencia, CA). For RNA analysis in HBEC, Affymetrix human gene 1.0-ST arrays were used. For expression profiles of HCT116-WT, HCT116-DKO, and HBEC non-targeting siRNA and HBEC SETD2 siRNA experiments, Affymetrix human exon 1.0-ST microarrays were applied. Microarrays were processed at the City of Hope microarray core facility. Arrays were scanned at 5 µm resolution using the Affymetrix GCS 3000 7G scanner and GeneChip Operating Software v. 1.4 to produce. CEL intensity files. Raw intensity measurements of all probe sets were background-corrected, normalized and converted into transcript or exon level expression measurements using the Affymetrix Expression Console v1.1.1. All data is MIAME compliant. The raw microarray data have been deposited in the Gene Expression Omnibus repository (accession number GSE26020), which is a MIAME compliant database, as detailed on the MGED Society website http://www.mged.org/Workgroups/MIAME/miame.html. Expression data were verified by real-time RT-PCR with cDNA obtained with iScript cDNA Synthesis Kit (BioRad).

### DNA analysis

DNA isolation was done with the DNAeasy kit according to the manufacturer's instructions (Qiagen). Bisulfite DNA conversion was done with the EZ DNA Methylation-Gold™ Kit (Zymo Research; Orange, CA). For DNA methylation analysis, bisulfite-modified DNA was amplified with FastTaq polymerase (Qiagen) and gene-specific primers (primer sequences are available on request). The amplicon was digested dependent on sequence with TaqIa, BstUI or HpyCH4IV (New England Biolabs; Beverly, MA), or cloned by using pGEMTeasy vector (Promega; Madison, WI) and TAM1 competent cells (Active Motif; Carlsbad, CA). After cloning, individual clones were sequenced.

MBD2B and MBD3L1 protein purification and MIRA were done as previously described [48]. For MIRA, sonicated genomic DNA was incubated with the MBD2B/MBD3L1 complex overnight at 4°C. The methylated DNA fraction was isolated by magnetic GST beads (Promega) and washed with MIRA washing buffer containing 700 mM NaCl. DNA was extracted by adding binding buffer from the PCR purification kit (Qiagen) and cleaned by using PCR purification kit according to the manufacturer's instructions. Profiling of unmethylated DNA was done with the UnMethylCollector kit (Active Motif; Carlsbad, CA) according to the manufacturer's instructions. This kit uses the CXXC domain of the MBD1 protein for enrichment of unmethylated DNA. Enriched unmethylated DNA and input fractions were processed for microarray hybridization similar as described for the MIRA protocol [48]. For genome amplification, DNA was incubated with T4 DNA polymerase (New England Biolabs) for 20 minutes at 12°C, purified with PCR purification kits and ligated with blunt end linker (5'-AGCAACTGTGCTATCCGAGGGAT and 5'-ATCCCTCGGA) overnight at 16°C with T4 ligase (New England Biolabs). Genome amplification was performed in the exponential phase with Taq polymerase (Qiagen). For MIRA quality control, real-time PCR with 10 ng amplicon and FastTaq polymerase (Qiagen) was done by using specific primers complementary to unmethylated gene promoters (*TBP*, *GAPDH*) or methylated targets (*H19*, *SNPRN*, *TGM3* gene body). Primer sequences are available on request. For DNA methylation and chromatin profiling, NimbleGen tiling microarrays of human chromosome 19 were used. Labeling, hybridization, and scanning of NimbleGen tiling arrays were performed at the City of Hope microarray core facility. The log2 ratios of MIRA signal vs. input were generated using NimbleScan v 5.0 (NimbleGen) with default settings. All microarray data is MIAME compliant. The raw microarray data have been deposited in the Gene Expression Omnibus repository (accession number GSE26020), which is a MIAME compliant database, as detailed on the MGED Society website http://www.mged.org/Workgroups/MIAME/miame.html.

### Chromatin immunoprecipitation (ChIP)

ChIP experiments were done as previously described [6]. In our experiments, we used the following antibodies: normal rabbit IgG (sc-2027, Santa Cruz Biotechnology, Inc.), anti-H3K36me3 (ab9050, Abcam), anti-H3K9me3 (07-442, Millipore), anti-acetyl-histone H3 (K9 and K14) (06-599, Millipore), anti-H3K4me3 (07-473, Millipore) and anti-H3K27me3 (07-449 Milipore). These antibodies have been verified and used previously in epigenome mapping studies [24], [25], [49], [50], [51], [52], [53].

### Profiles at the gene body and promoter

The hg18 RefSeq database (http://hgdownload.cse.ucsc.edu/goldenPath/hg18/database/) was used for annotation. The promoter region was defined as +/−500 bp relative to the transcription start site, and ‘gene body’ was defined as from the transcription start site to the end of the transcript. For DNA methylation and histone modifications, the average log2 ratios of probes falling into promoters or gene bodies were calculated. For CpG count in the gene body, the number of CpG dinucleotides within gene bodies was counted and divided by the length of the gene body and then scaled to counts per 1 kb.

### Gene body methylation and histone mark coverage

The probes with log2 ratio >1 were considered as positive. The gene body coverage for each gene in the hg18 RefSeq database was represented by the percentage of positive probes within its gene body. To identify the genes covered by multiple marks, the genes with > = 20% positive probe coverage for each mark were identified and then the gene symbols were matched across multiple marks.

### Composite profiles

The gene body for each RefSeq gene was divided into 20 equal-sized bins, and its 5 kb upstream and downstream region was divided into 10 equal-sized bins each. For each bin of each gene, the probes falling into the bin were retrieved and their log2 ratio for each marker was averaged to represent its signal within that bin of the gene. For each bin, the average probe signal of each marker for all the genes are then averaged and plotted. The signals were used either for heatmap directly (Figure 1) or averaged for composite profile generation (e.g., Figure 3D).

### Exon-level analysis

Exon level intensity data were obtained using the RMA method of Partek Genomic Suite v6.5 (Partek; St. Louis, MO), and those located on chr19 were kept for subsequent analysis. The top 15% exons with the highest expression level and bottom 15% exons with the lowest expression level were selected based on their log2 intensity distribution, and denoted as exons with high and low expression, respectively. For the genes covered with either H3K36m3 and MIRA signal or with H3K36m3 and H3K9m3 signal, the average log2 ratio signal of the probes falling into the two expression categories were calculated. For genes with at least two exons, the average log2 ratio signal of the probes falling into the first exon, last exon, and each intron were calculated. For the genes on chr19 with at least two coding exons, the average log2 ratio signal of the probes falling into the first coding exon and last coding exon were calculated. The distribution of these average probe signals was presented as boxplots (Figure S4).

### Gene ontology analysis

The gene lists were uploaded to DAVID functional annotation tools (http://david.abcc.ncifcrf.gov/) and genes on chr19 were used as background.

## Supporting Information

Figure S1 **Interrelationship between DNA methylation and the H3K36me3 modification.** **A**. The DNA methylation level is dropping after the 3′ gene end of an H3K36me3-occupied gene. The DNA methylation status of the 3′ gene end and the neighboring region was analyzed for the *LSM* gene in HBEC cells. DNA methylation and H3K36me3 microarray profiles are shown. Direction of transcription, gene coordinates and locations of the COBRA-analyzed regions (blue boxes) are indicated. Using gene-specific primers, bisulfite-converted DNA was amplified. After cutting with TaqIa, recognizing CpG dinucleotides, mock (-) and enzyme-digested (+) PCR products were separated on a 2% agarose gel. In vitro CpG-methylated human DNA (M) served as a positive control. Cleavage indicates DNA methylation. **B**. COBRA analysis of an intragenic region of the *POLD1* gene showing DNA methylation and H3K36me3 occupancy. The analyzed region is indicated by a blue box. **C**. COBRA analysis of an intragenic region of the *NFKBIB* gene showing DNA methylation and H3K36me3 occupancy. The analyzed region is indicated by a blue box. **D**. Negative correlation between unmethylated DNA (UMC) and H3K36me3 in HBEC. Averages of UMC signal in the gene body for each gene were spotted versus average of H3K36me3 signal in the gene body. **E**. Positive correlation between methylated DNA (MIRA) and H3K36me3 in HBEC. Averages of MIRA signal in the gene body of each gene were spotted versus average of H3K36me3 signal in the gene body. **F**. Negative correlation between histone H3 acetylation (H3Ac) and DNA methylation (MIRA) in promoters (-1000 to +500 bp of the transcription start sites). **G**. Negative correlation between unmethylated DNA signal (UMC) and methylated DNA signal (MIRA) in promoters.
(PDF)

Figure S2 **Crosstalk between DNA methylation and H3K27me3 in HBEC.** **A**. Gene bodies marked by DNA methylation and H3K27me3 on human chr19 in HBEC. The numbers indicate genes with gene bodies marked by H3K27me3 or DNA methylation or by both marks with at least 20% of gene body length coverage (p<0.001; Chi square test). **B**. Composite profile of genes with gene bodies marked by H3K27me3 and DNA methylation with at least 20% of gene body length coverage. Each gene body was divided into 20 bins and the 5 kb upstream of the TSS and 5 kb downstream of the 3′ gene end were divided into 10 bins. The average signal for each single bin is plotted. Both MIRA and UMC signals are enriched within H3K27me3-covered gene bodies indicating partial methylation. **C**. Representative epigenetic profile of a chr19 region containing H3K27me3-marked genes (boxes) in HBEC. **D**. DNA methylation level in gene bodies of different epigenetic gene groups, genes marked by H3K27me3 together with DNA methylation, genes marked by H3K9me3 and H3K36me3, and genes marked by DNA methylation together with H3K36me3. The average of signal in each gene body was plotted for each epigenetic group. A gene was considered marked by a specific epigenetic modification if this mark was present along at least 20% of gene body length. Multiple comparison tests show that all three pairs show statistical difference at p<0.001. **E**. CpG density in gene bodies for genes marked by H3K27me3 and DNA methylation, by H3K9me3 and H3K36me3, and by DNA methylation together with H3K36me3. Average of CpG density for each gene body was plotted for each epigenetic group. Comparison between H3K27me3-MIRA and H3K36me3-H3K9me3 shows statistical difference (p<0.001). Comparison between H3K36me3-MIRA and H3K36me3-H3K9me3 also shows statistical difference (p<0.001). There is no statistical difference between H3K27me3-MIRA and H3K36me3-MIRA (p>0.05).
(PDF)

Figure S3 **Epigenetic characteristics of H3K9me3-associated genes on chr19 in HBEC.** **A**. Partial DNA methylation in gene bodies of genes occupied with H3K9me3. A representative epigenetic profile of an H3K9me3-enriched gene is shown. Direction of transcription, gene coordinates and the region analyzed by COBRA in the *NAPL1* gene are indicated. DNA methylation analysis was performed by COBRA for three CpG-poor and H3K9me3-occupied genes (*NALP1*, *UNC13A*, and *ZNF536*) with partial DNA methylation as indicated by UMC signal. In vitro CpG-methylated human DNA (M) served as a positive control. Cleavage indicates DNA methylation. **B**. Gene ontology terms associated with H3K9me3- and H3K36me3 dual-occupied genes according to analysis via DAVID.
(PDF)

Figure S4 **Gene body DNA modifications and chromatin marks in relation to exon and intron structure.** Genes on chromsome 19 were subdivided into two categories. Panels **A** and **B** show genes co-occupied with H3K36me3 and DNA methylation. Panels **C** and **D** represent genes co-occupied with H3K36me3 and H3K9me3. Using data from Affymetrix exon arrays, we determined which exons are expressed at low levels (bottom 15% of expressed genes) and which ones are expressed at high levels (top 15% of expressed genes). The MIRA, H3K36m3 or H3K9m3 average signal (log2 ratio) of probes in the selected exons or introns is plotted. We also plotted the signals for the first and last exons and the first and last coding exons, respectively. **A**. H3K36me3 levels in H3K36me3 and DNA methylation co-occupied genes. **B** DNA methylation levels in H3K36me3 and DNA methylation co-occupied genes. **C**. H3K36me3 levels in H3K36me3 and H3K9me3 co-occupied genes. **A**. H3K9me3 levels in H3K36me3 and H3K9me3 co-occupied genes.
(PDF)

Figure S5 **DNA methylation and epigenetic profiles in HCT116-DKO and HCT116-WT cells.** **A**. DNA methylation in LINE1 elements in HCT116-DKO and HCT116-WT cells. Using LINE1-promoter-specific primers, bisulfite-converted DNA was amplified. After cutting with HinfI, recognizing CpG dinucleotides, mock (-) and enzyme-digested (+) PCR products were fractionated by size on a 2% agarose gel. In vitro CpG-methylated human DNA (M) served as a positive control. Cleavage indicates DNA methylation, which is almost completely lost in DKO cells **B**. MIRA and H3K36me3 distribution profile along the *DNMT1* gene in HCT116-DKO and HCT116-WT cells. Note the loss of signal between exons 3 and 5 (yellow box) in DKO cells due to gene disruption but persistent transcription as indicated by the H3K36me3 profile. **C**. Verification of retained DNA methylation in some gene bodies in HCT116-DKO cells. Representative profile of persistent DNA methylation in gene bodies in HCT116-DKO cells and comparison to the profile in HCT116-WT cells. Red, DNA methylation; green, H3K36me3. The gene coordinates and location of the analyzed region in the *NFKBIB* gene are indicated. DNA methylation analysis was performed by COBRA for two different genes retaining DNA methylation in the gene body according to the MIRA data. Using gene-specific primers, bisulfite-converted DNA was amplified. After cutting with TaqIa, recognizing CpG dinucleotides, mock (-) and enzyme-digested (+) PCR products were fractionated by size on a 2% agarose gel. In vitro CpG-methylated human DNA (M) served as a positive control. Cleavage indicates DNA methylation.
(PDF)

Figure S6 **Epigenetic profiles of H3K9me3-associated genes that are either upregulated or downregulated in HCT116-DKO cells.** **A**. Composite profile of upregulated H3K9me3-covered genes in HCT116-WT and DKO cells. The profile was created for genes with gene bodies covered by H3K9me3 with at least 20% of gene body length coverage in HCT116-WT cells and upregulated transcription by at least log2 of 0.5 in comparison to HCT116-WT cells. **B**. Composite profile of downregulated H3K9me3 genes in HCT116-DKO. The profile was created for genes with gene bodies covered by H3K9me3 with at least 20% of gene body length coverage in HCT116-WT cells and downregulated transcription by at least log2 of 0.5 in comparison to HCT116-WT cells.
(PDF)
