# Supplementary figures and images for: Relationship between Gene Body DNA Methylation and Intragenic H3K9me3 and H3K36me3 Chromatin Marks
Source: PLoS One. 2011 Apr 19;6(4):e18844. doi: 10.1371/journal.pone.0018844 (PMC3079728; doi:10.1371/journal.pone.0018844)

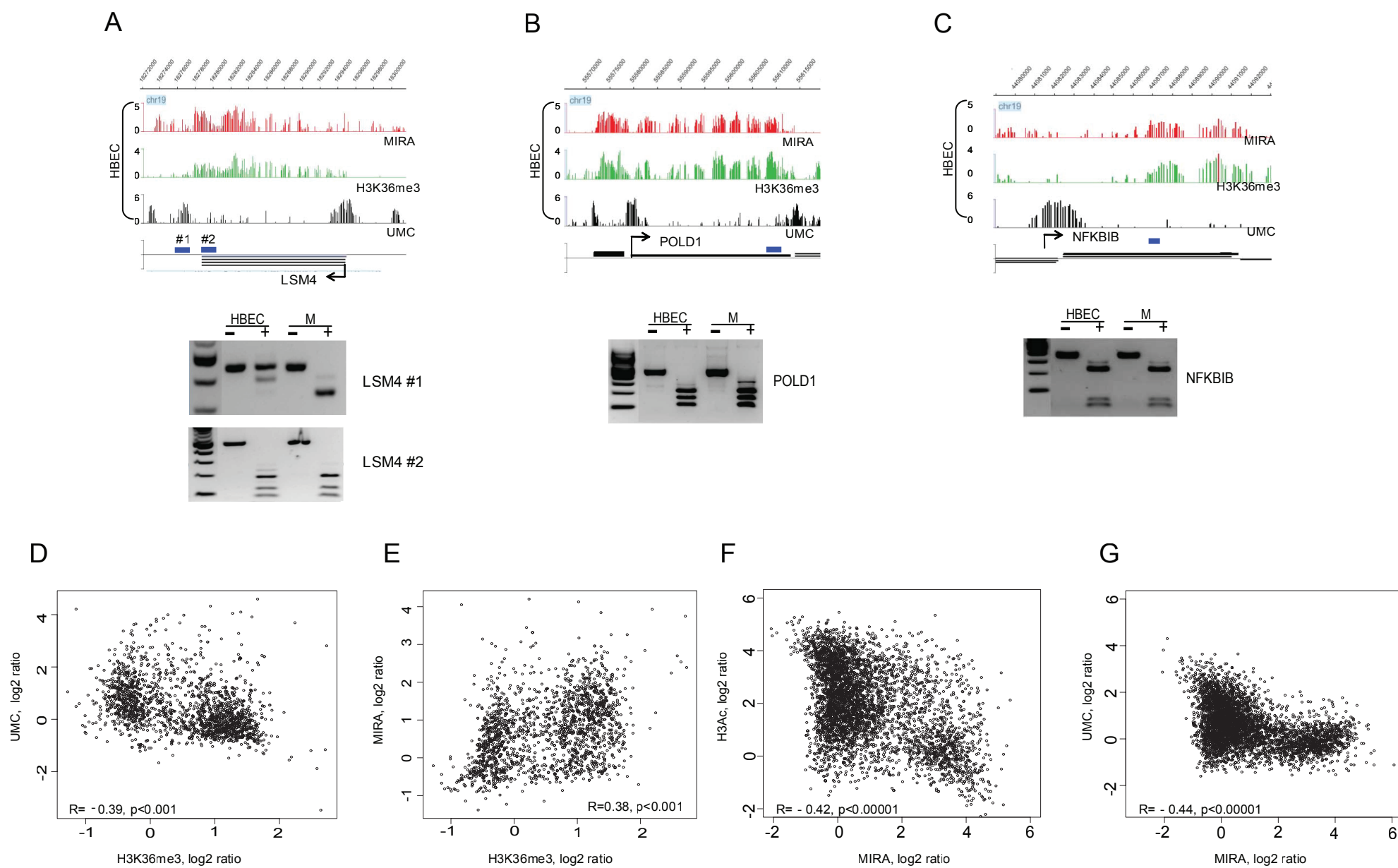

Suppl. Figure 1

Supplement: Figure S1 — Interrelationship between DNA methylation and the H3K36me3 modification. A. The DNA methylation level is dropping after the 3′ gene end of an H3K36me3-occupied gene. The DNA methylation status of the 3′ gene end and the neighboring region was analyzed for the LSM gene in HBEC cells. DNA methylation and H3K36me3 microarray profiles are shown. Direction of transcription, gene coordinates and locations of the COBRA-analyzed regions (blue boxes) are indicated. Using gene-specific primers, bisulfite-converted DNA was amplified. After cutting with TaqIa, recognizing CpG dinucleotides, mock (-) and enzyme-digested (+) PCR products were separated on a 2% agarose gel. In vitro CpG-methylated human DNA (M) served as a positive control. Cleavage indicates DNA methylation. B. COBRA analysis of an intragenic region of the POLD1 gene showing DNA methylation and H3K36me3 occupancy. The analyzed region is indicated by a blue box. C. COBRA analysis of an intragenic region of the NFKBIB gene showing DNA methylation and H3K36me3 occupancy. The analyzed region is indicated by a blue box. D. Negative correlation between unmethylated DNA (UMC) and H3K36me3 in HBEC. Averages of UMC signal in the gene body for each gene were spotted versus average of H3K36me3 signal in the gene body. E. Positive correlation between methylated DNA (MIRA) and H3K36me3 in HBEC. Averages of MIRA signal in the gene body of each gene were spotted versus average of H3K36me3 signal in the gene body. F. Negative correlation between histone H3 acetylation (H3Ac) and DNA methylation (MIRA) in promoters (-1000 to +500 bp of the transcription start sites). G. Negative correlation between unmethylated DNA signal (UMC) and methylated DNA signal (MIRA) in promoters. (PDF) [file pone.0018844.s001.pdf]

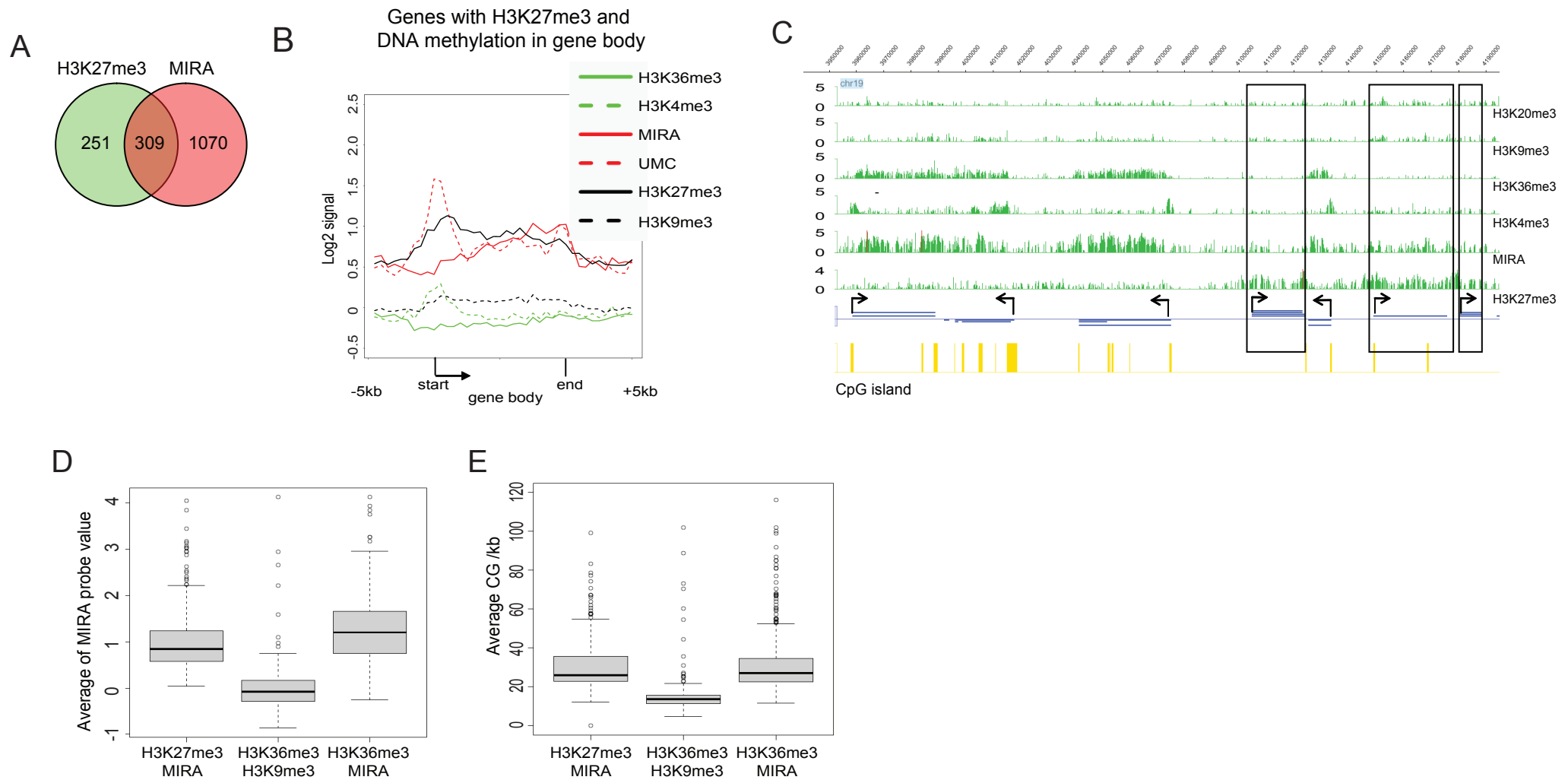

Suppl. Fig. 2

Supplement: Figure S2 — Crosstalk between DNA methylation and H3K27me3 in HBEC. A. Gene bodies marked by DNA methylation and H3K27me3 on human chr19 in HBEC. The numbers indicate genes with gene bodies marked by H3K27me3 or DNA methylation or by both marks with at least 20% of gene body length coverage (p<0.001; Chi square test). B. Composite profile of genes with gene bodies marked by H3K27me3 and DNA methylation with at least 20% of gene body length coverage. Each gene body was divided into 20 bins and the 5 kb upstream of the TSS and 5 kb downstream of the 3′ gene end were divided into 10 bins. The average signal for each single bin is plotted. Both MIRA and UMC signals are enriched within H3K27me3-covered gene bodies indicating partial methylation. C. Representative epigenetic profile of a chr19 region containing H3K27me3-marked genes (boxes) in HBEC. D. DNA methylation level in gene bodies of different epigenetic gene groups, genes marked by H3K27me3 together with DNA methylation, genes marked by H3K9me3 and H3K36me3, and genes marked by DNA methylation together with H3K36me3. The average of signal in each gene body was plotted for each epigenetic group. A gene was considered marked by a specific epigenetic modification if this mark was present along at least 20% of gene body length. Multiple comparison tests show that all three pairs show statistical difference at p<0.001. E. CpG density in gene bodies for genes marked by H3K27me3 and DNA methylation, by H3K9me3 and H3K36me3, and by DNA methylation together with H3K36me3. Average of CpG density for each gene body was plotted for each epigenetic group. Comparison between H3K27me3-MIRA and H3K36me3-H3K9me3 shows statistical difference (p<0.001). Comparison between H3K36me3-MIRA and H3K36me3-H3K9me3 also shows statistical difference (p<0.001). There is no statistical difference between H3K27me3-MIRA and H3K36me3-MIRA (p>0.05). (PDF) [file pone.0018844.s002.pdf]

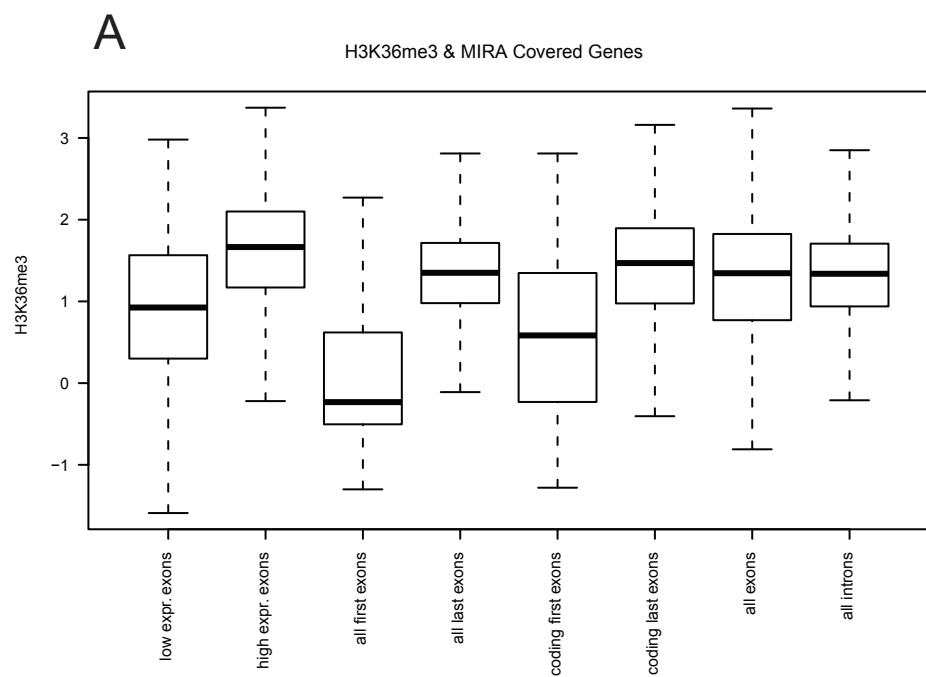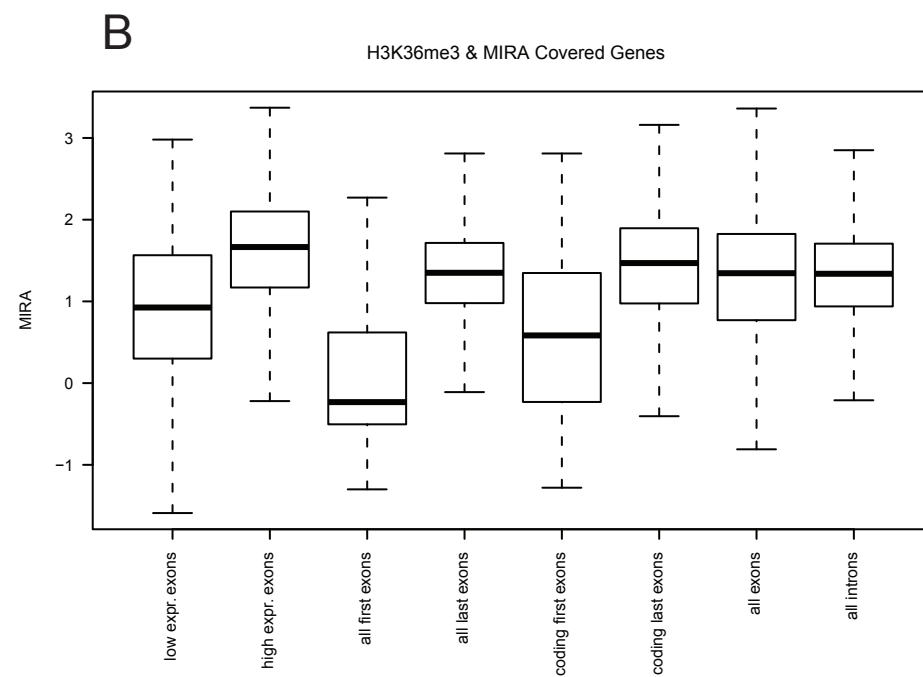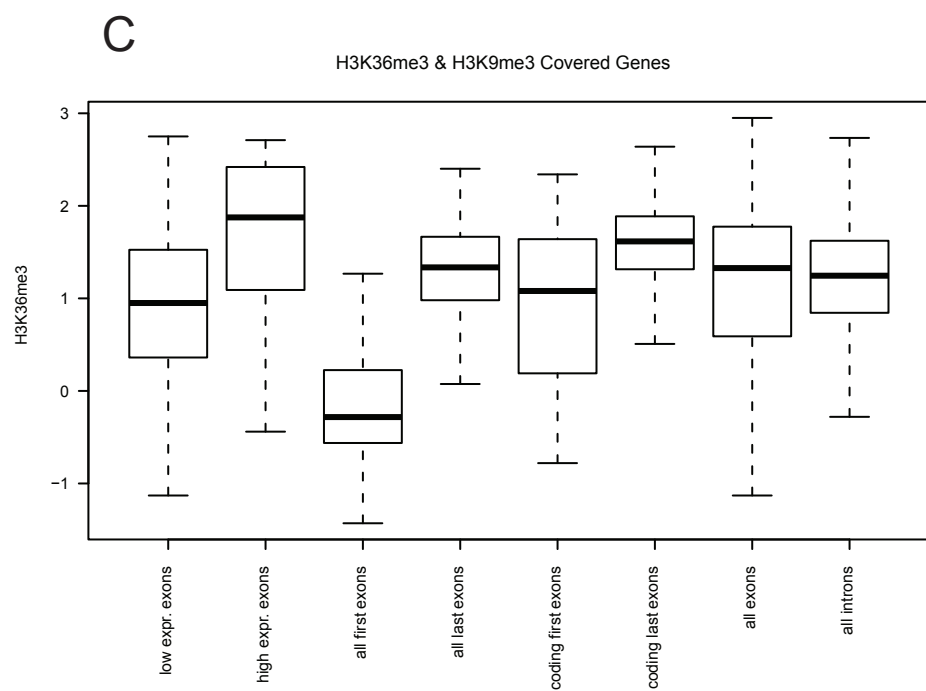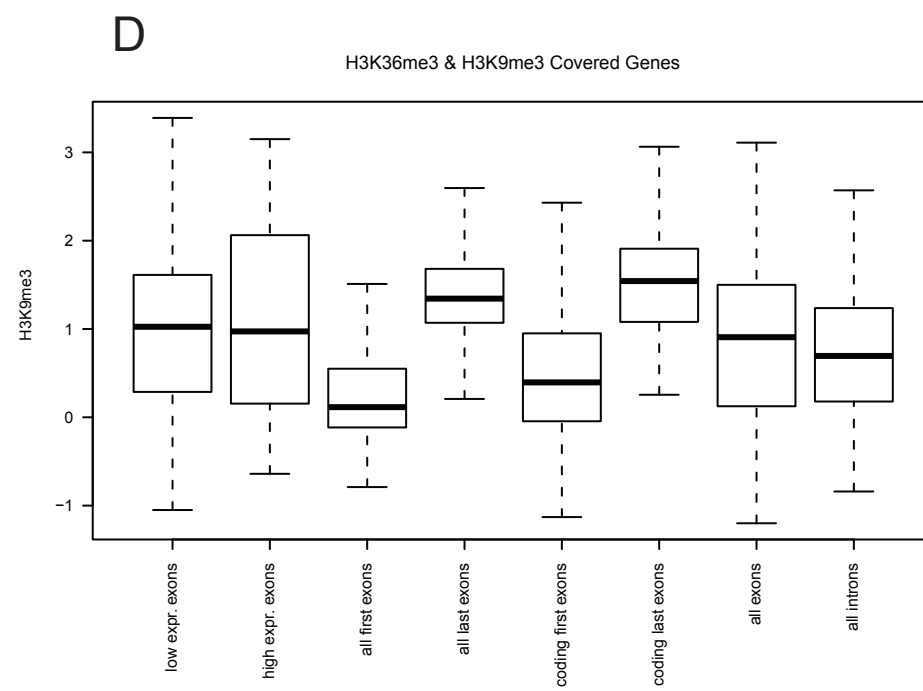

Supplement: Figure S4 — Gene body DNA modifications and chromatin marks in relation to exon and intron structure. Genes on chromsome 19 were subdivided into two categories. Panels A and B show genes co-occupied with H3K36me3 and DNA methylation. Panels C and D represent genes co-occupied with H3K36me3 and H3K9me3. Using data from Affymetrix exon arrays, we determined which exons are expressed at low levels (bottom 15% of expressed genes) and which ones are expressed at high levels (top 15% of expressed genes). The MIRA, H3K36m3 or H3K9m3 average signal (log2 ratio) of probes in the selected exons or introns is plotted. We also plotted the signals for the first and last exons and the first and last coding exons, respectively. A. H3K36me3 levels in H3K36me3 and DNA methylation co-occupied genes. B DNA methylation levels in H3K36me3 and DNA methylation co-occupied genes. C. H3K36me3 levels in H3K36me3 and H3K9me3 co-occupied genes. A. H3K9me3 levels in H3K36me3 and H3K9me3 co-occupied genes. (PDF) [file pone.0018844.s004.pdf]

A

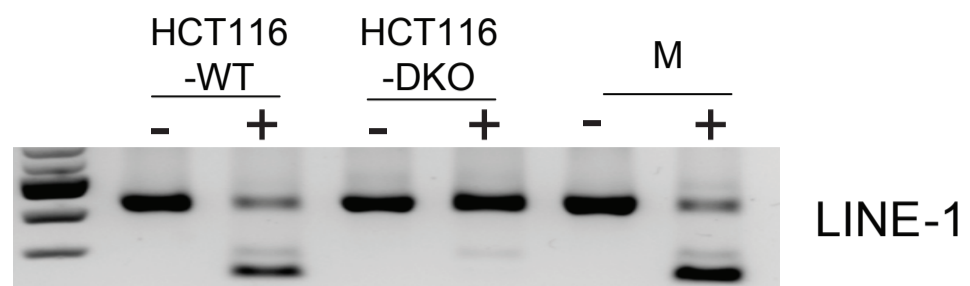

C

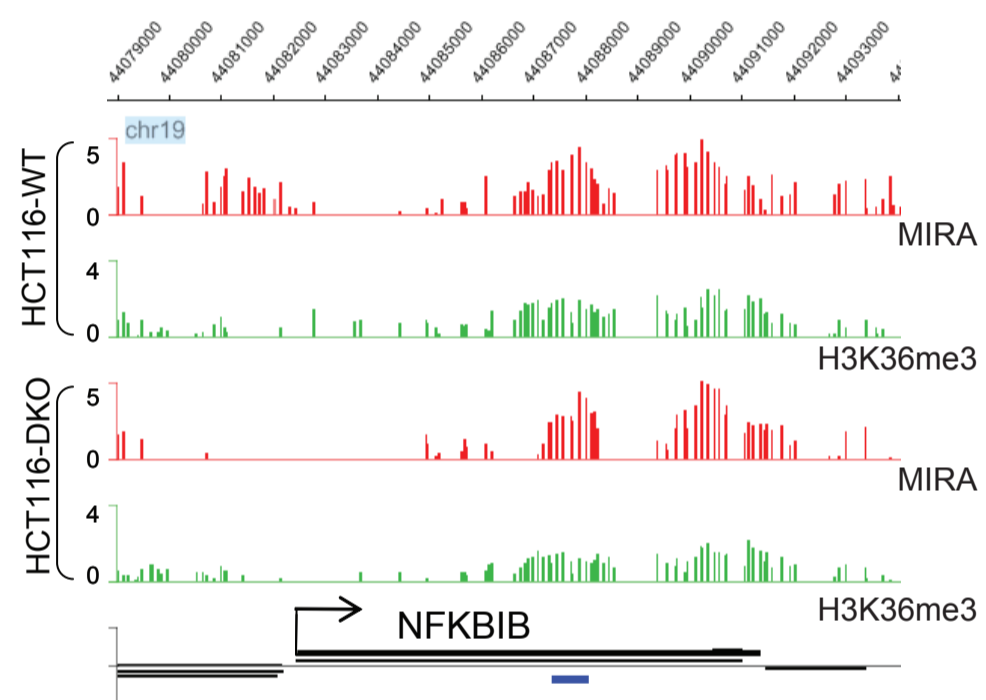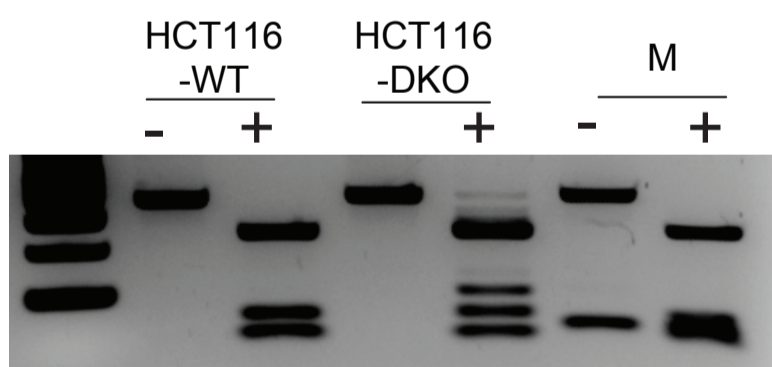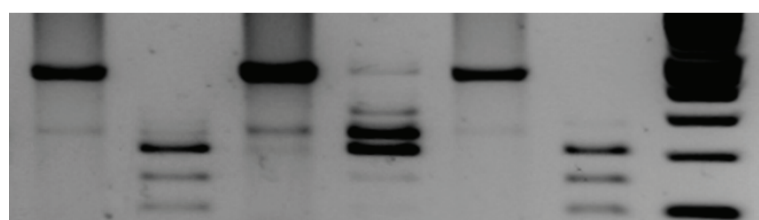

B

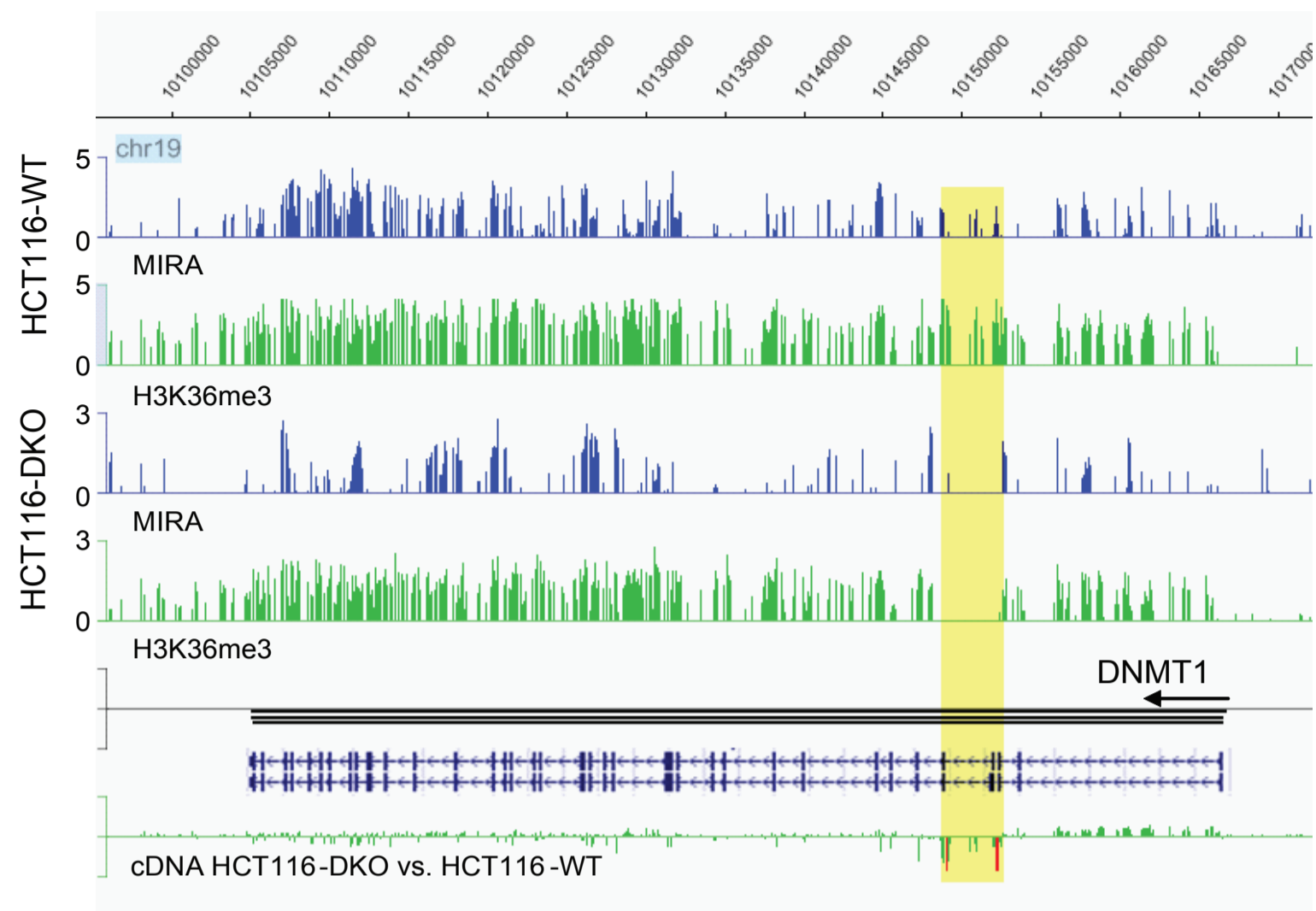

Suppl. Figure 5

Supplement: Figure S5 — DNA methylation and epigenetic profiles in HCT116-DKO and HCT116-WT cells. A. DNA methylation in LINE1 elements in HCT116-DKO and HCT116-WT cells. Using LINE1-promoter-specific primers, bisulfite-converted DNA was amplified. After cutting with HinfI, recognizing CpG dinucleotides, mock (-) and enzyme-digested (+) PCR products were fractionated by size on a 2% agarose gel. In vitro CpG-methylated human DNA (M) served as a positive control. Cleavage indicates DNA methylation, which is almost completely lost in DKO cells B. MIRA and H3K36me3 distribution profile along the DNMT1 gene in HCT116-DKO and HCT116-WT cells. Note the loss of signal between exons 3 and 5 (yellow box) in DKO cells due to gene disruption but persistent transcription as indicated by the H3K36me3 profile. C. Verification of retained DNA methylation in some gene bodies in HCT116-DKO cells. Representative profile of persistent DNA methylation in gene bodies in HCT116-DKO cells and comparison to the profile in HCT116-WT cells. Red, DNA methylation; green, H3K36me3. The gene coordinates and location of the analyzed region in the NFKBIB gene are indicated. DNA methylation analysis was performed by COBRA for two different genes retaining DNA methylation in the gene body according to the MIRA data. Using gene-specific primers, bisulfite-converted DNA was amplified. After cutting with TaqIa, recognizing CpG dinucleotides, mock (-) and enzyme-digested (+) PCR products were fractionated by size on a 2% agarose gel. In vitro CpG-methylated human DNA (M) served as a positive control. Cleavage indicates DNA methylation. (PDF) [file pone.0018844.s005.pdf]

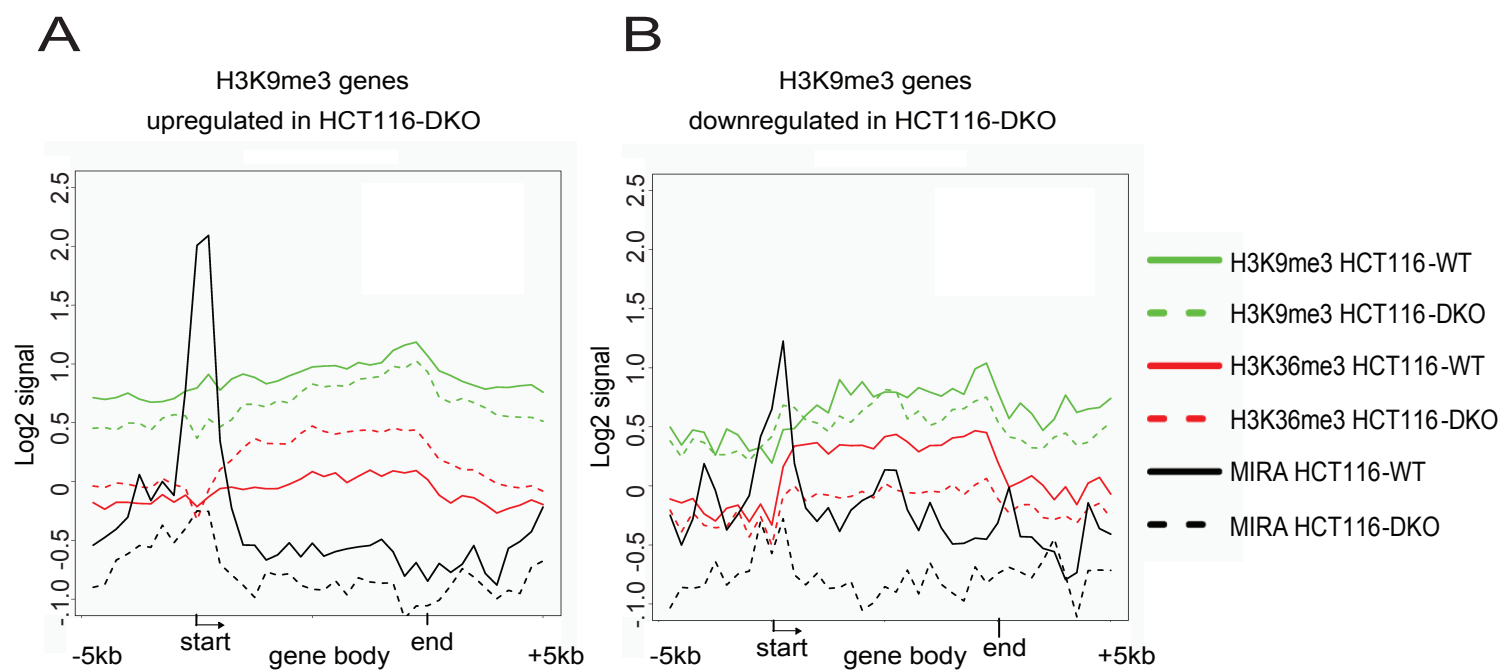

Suppl. Figure 6

Supplement: Figure S6 — Epigenetic profiles of H3K9me3-associated genes that are either upregulated or downregulated in HCT116-DKO cells. A. Composite profile of upregulated H3K9me3-covered genes in HCT116-WT and DKO cells. The profile was created for genes with gene bodies covered by H3K9me3 with at least 20% of gene body length coverage in HCT116-WT cells and upregulated transcription by at least log2 of 0.5 in comparison to HCT116-WT cells. B. Composite profile of downregulated H3K9me3 genes in HCT116-DKO. The profile was created for genes with gene bodies covered by H3K9me3 with at least 20% of gene body length coverage in HCT116-WT cells and downregulated transcription by at least log2 of 0.5 in comparison to HCT116-WT cells. (PDF) [file pone.0018844.s006.pdf]
