# Supplementary material for: Relationship between Gene Body DNA Methylation and Intragenic H3K9me3 and H3K36me3 Chromatin Marks
Source: PLoS One. 2011 Apr 19;6(4):e18844. doi: 10.1371/journal.pone.0018844 (PMC3079728; doi:10.1371/journal.pone.0018844)
Supplement: Figure S3 — Epigenetic characteristics of H3K9me3-associated genes on chr19 in HBEC. A. Partial DNA methylation in gene bodies of genes occupied with H3K9me3. A representative epigenetic profile of an H3K9me3-enriched gene is shown. Direction of transcription, gene coordinates and the region analyzed by COBRA in the NAPL1 gene are indicated. DNA methylation analysis was performed by COBRA for three CpG-poor and H3K9me3-occupied genes (NALP1, UNC13A, and ZNF536) with partial DNA methylation as indicated by UMC signal. In vitro CpG-methylated human DNA (M) served as a positive control. Cleavage indicates DNA methylation. B. Gene ontology terms associated with H3K9me3- and H3K36me3 dual-occupied genes according to analysis via DAVID. (PDF) [file pone.0018844.s003.pdf]

A

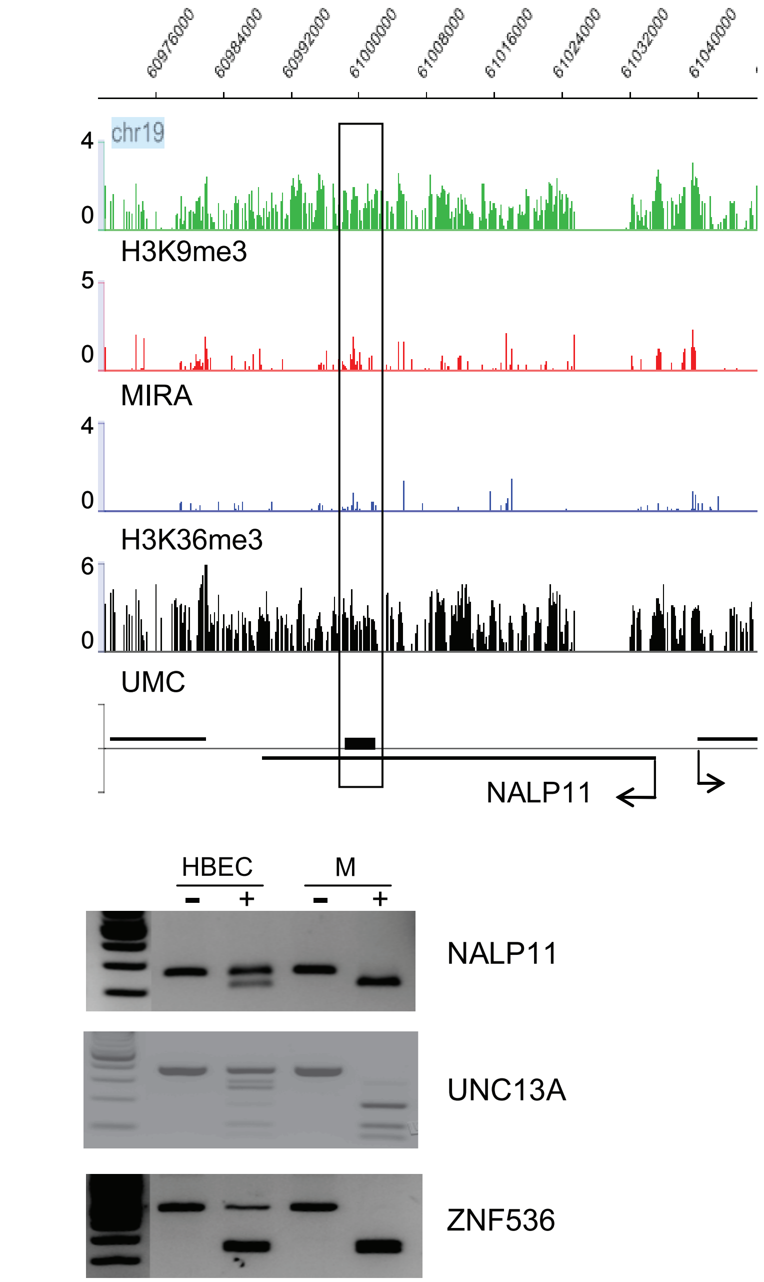

B

Term	RT	Genes	Count	%	P-Value	Benjamini
zinc finger region:C2H2-type 7	RT		148	55.6	1.5E-180	8.4E-178
zinc finger region:C2H2-type 8	RT		142	53.4	9.6E-175	2.7E-172
zinc finger region:C2H2-type 6	RT		147	55.3	1.0E-172	1.9E-170
zinc finger region:C2H2-type 9	RT		135	50.8	1.5E-168	2.1E-166
domain:KRAB	RT		130	48.9	2.0E-167	2.2E-165
zinc finger region:C2H2-type 5	RT		146	54.9	9.6E-164	9.1E-162
zinc finger region:C2H2-type 10	RT		126	47.4	4.1E-159	3.3E-157
zinc finger region:C2H2-type 3	RT		148	55.6	6.3E-157	4.5E-155
zinc finger region:C2H2-type 4	RT		144	54.1	1.9E-155	1.2E-153
zinc finger region:C2H2-type 11	RT		112	42.1	2.9E-140	1.6E-138
zinc finger region:C2H2-type 2	RT		131	49.2	9.6E-130	5.0E-128
zinc finger region:C2H2-type 12	RT		101	38.0	9.8E-128	4.7E-126
zinc finger region:C2H2-type 13	RT		83	31.2	1.2E-104	5.4E-103
zinc finger region:C2H2-type 14	RT		70	26.3	3.3E-90	1.3E-88
zinc finger region:C2H2-type 15	RT		64	24.1	1.6E-84	5.9E-83
zinc finger region:C2H2-type 1	RT		97	36.5	5.0E-84	1.8E-82
transcription	RT		167	62.8	5.1E-83	1.9E-80
zinc finger region:C2H2-type 1; dege	RT		60	22.6	5.4E-80	1.8E-78

Suppl. Fig. 3
